# Development and Evaluation of a Digital Health Intervention for Substance Use Reduction in Young Refugees With Problematic Use of Alcohol and/or Cannabis—Study Protocol for a Single-Armed Feasibility Trial

**DOI:** 10.3389/fpubh.2021.557431

**Published:** 2021-03-31

**Authors:** Laura Charlotte Fischer, Vera Kölligan, Nuri Wieland, Michael Klein

**Affiliations:** German Institute on Addiction and Prevention Research (DISuP), Catholic University of Applied Sciences of North Rhine–Westphalia, Cologne, Germany

**Keywords:** refugees, digital health intervention, indicated prevention, problematic substance use, brief intervention, cultural adaptation, addiction

## Abstract

**Background:** Refugee populations are at substantial risk of developing substance use disorder (SUD) and other mental health disorders. At the same time, refugee populations face numerous barriers to accessing mental health services. Digital interventions can address some of these issues, as emerging evidence indicates that digital interventions offer an effective, low-cost alternative with high accessibility and similar efficacy as standard SUD prevention programs. As an add-on to traditional services, they further present a viable approach to the lack of personnel available for foreign language communication in preventive and therapeutic settings. In the present study, we thus aim to develop and evaluate a digital health intervention (DHI) for the reduction of substance use for young refugees with problematic use of alcohol and/or cannabis. The intervention will be implemented in a smartphone app, translated into Arabic, English, Farsi, German, and Pashto, and is to be used stand-alone. It is based on methods from cognitive behavioral therapy, contains culturally adapted elements, provides practical information on how to deal safely with alcohol and cannabis, and motivates behavior change through increased self-reflection and behavioral, cognitive, and emotional skills-training in interactive exercises.

**Methods:** We conduct a single-armed feasibility trial among 150 young refugees with problematic use of alcohol and/or cannabis. Participants will receive a digital screening and digital health intervention (DHI) for the reduction of problematic substance use, carried out over a 4-week time frame. The primary outcomes are the intervention's feasibility and the target population's acceptance of the intervention. The secondary outcome is a change in substance use post-intervention from baseline. Measurements are taken pre-intervention (baseline), post-intervention (after 4 weeks), and at 3- and 6-month follow-ups. We expect the intervention to be feasible and accepted by the target group.

**Discussion:** The present study will establish to what degree the digital intervention (the “BePrepared App”) is feasible and accepted by the target group. The evaluation of an easily accessible, feasible, and accepted intervention may impact refugees' mental health and health-related consequences. It aims at overcoming barriers to preventive health care in the substance use field for underserved refugee populations as a tool within a larger set of urgently needed interventions.

**Trial Registration:** DRKS00021095 at the “German Clinical Trials Register” (30.03.2020).

## Background

In the end of 2019, the United Nations High Commissioner for Refugees (UNHCR) estimated a number of 79.5 million forcibly displaced people, i.e., people who are driven from their homes by persecution, conflict, violence, human rights violations, or events seriously disturbing public order. This is a record high, which accelerated in recent years as a response to the armed conflicts in Syria, but also conflicts in other areas like the Middle East, parts of sub-Saharan Africa, and the persecution of the Rohingya people in Myanmar (1). In 2019, the largest single group of people asking for asylum in Germany were of Syrian origin (27.3%, *n* = 39.270), followed by people of Iraqi origin (9.5%, *n* = 13.742) ranked second, Turkish origin (7.4%, *n* = 10.784) ranked third, Afghan origin (6.7%, *n* = 9.522) ranked fourth, Nigerian origin (6.2%, *n* = 9.070) ranked fifth, and Iranian origin (5.7%, *n* = 8.407) ranked sixth (2). On the basis of the 1951 Refugee Convention, a refugee is protected by international law and defined as “someone who is unable or unwilling to return to their country of origin owing to a well-founded fear of being persecuted for reasons of race, religion, nationality, membership of a particular social group, or political opinion” [(3), p. 3]. On their website, the UNHCR further describes refugees as “people who have fled war, violence, persecution and have crossed an international border to find safety in another country” [(4), para. 1]. For ease of reading, we will use the word *refugee* interchangeably with the terms *forcibly displaced people* and *forced migrants*.

There is an urgent need to support the mental health of refugees (5), who are in a particularly vulnerable situation due to exposure to trauma and other adverse health effects before migrating from their homes in conflict-affected settings, during their journey, and upon arrival in intermediate and final receiving countries (6, 7). Though estimates regarding refugees' mental health vary widely, a growing number of studies and meta-analyses suggest an elevated risk for mental health disorders in refugees [for an overview see Henkelmann et al. (8), Silove et al. (9), and Crepet et al. (10)]. New WHO estimates adjust previous estimates upwards, showing a high prevalence of mental health disorders among people affected by conflict and war. Of these, one person in five (22.1%) lives with some form of depression, anxiety disorder, post-traumatic stress disorder (PTSD), bipolar disorder, or schizophrenia, compared to the general population in which “only” one person in 14 with any mental disorder worldwide does (11). The Federal Chamber of Psychotherapists in Germany (12) estimated in 2015 that 40–50% of refugees in Germany suffer from PTSD or depression. Of the (mostly West African) refugees who arrived in Italy in 2015, ~89% experienced possibly traumatic events during their often partly seaborne migration (10). Less data on experienced trauma is available for refugees arriving to Greece crossing the Mediterranean Sea, the majority of refugees having fled Syria (57%), Afghanistan (22%), and Iraq (5%) (until mid 2015) and most of which wanting to settle in Germany or Sweden (13).

In the general population, evidence from longitudinal (14–16) and experimental studies (17, 18) suggests that certain mental health disorders, such as PTSD, previous alcohol or drug use, depressive or anxiety disorders, predict later alcohol abuse or dependence and illicit drug abuse and dependence. The Diagnostic and Statistical Manual of Mental Disorders, Fifth Edition (DSM-5), merges *substance abuse* and *substance dependence* into a single classification of *substance use disorder* (SUD). On a continuum from mild to severe, it describes “a cluster of cognitive, behavioral, and physiological symptoms indicating that the individual continues using the substance despite significant substance-related problems” [(19), p. 483]. *Problematic, harmful, or hazardous substance use* describes an absence of a current substance-related disorder, however, increases the risk of harmful consequences, including physical, mental, and social problems for the user or others (20). It predicts the development of substance use related harm such as SUD, social problems, and the utilization of health care services (21).

According to the United Nations Office on Drug and Crime World Drug Report (22), risk factors for substance use are a refugee status, poverty, conflict/war, homelessness, social exclusion and inequality, neighborhood disorders, peer substance use and drug availability, mental health problems, trauma and childhood adversity. Problematic use of substances and the transition to SUD are known to have a higher prevalence in people with a lower socioeconomic status [SES; e.g., Legleye et al. (23)] as well as in people with certain mental disorders (14–18).

Initial research suggests that displacement experiences, their potential consequences and conditions in hosting countries (e.g., uncertain future prospects) may increase vulnerability to substance use, its impact and the transition to more harmful patterns of substance use (24). From an epidemiological viewpoint, substance use and its harmful consequences are common among refugee populations. For example, systematic reviews identify prevalences of problematic alcohol use between 17 and 36% in camp settings and 4–7% in community settings (25) and high levels of opioid use due to forced displacement that is caused by armed conflict in Pakistani and Afghan populations (26). Initial research on the drug use and special needs of refugees in Germany shows that 28.4% of a sample of predominantly Iranian and Afghan refugees interviewed in low-threshold addiction services have started using alcohol and/or illicit drugs *after* leaving their home country. 16.4% of the interviewed people were occasionally consuming alcohol and cannabis, the most-used substance being opioids (27). According to qualitative research based on expert interviews, substance-using unaccompanied minor refugees are primarily using alcohol or cannabis and have started using after arrival in Germany (28). Although remaining under the national average, data from the transcultural psychiatric department in Vienna, Austria, shows an elevated alcohol consumption from patients with muslim background from the middle east, compared to the average consumption in their countries of origin (29). The opposite was shown for cannabis, where the reported consumption remained under the expected value, based on the comsumption pattern in the countries of origin. German law prohibits cannabis acquisition and possession while alcohol remains legal for adults. Increased alcohol consumption and decreased cannabis consumption among refugees might be heavily influenced by legal regulations and social norms in the country of stay. Generally, it was found that regardless of former protective factors such as religious background, consumption patterns adapt to the social majority ones when length of stay increases [e.g., Arfken et al. (30); Harris et al. (31)]. It still remains questionable to what extend protective effects of cultural and social norms can be attributed to the subgroup of forced migrants (25). Next to research on protective factors, further research is required to assess key risk factors, patterns of use, and the extent of conflict-associated harmful use of e.g., alcohol beyond a merely suggestive causal relationship (32, 33).

These results, including the high number of Iranian and Afghan refugees who have started using alcohol and illegal substances after leaving their home country or upon arrival in Germany and, furthermore, the elevated risk for a transition to more harmful patterns of substance use, highlight the need for low-threshold early and indicated prevention targeting (young) refugees; Especially since asylum seekers in Germany tend to become younger over the last 5 years, with a growing majority being underage and a second largest group being between 18 and 25 years old (34). Indicated prevention describes interventions in individuals who show a manifest risky or problematic behavior (35), such as problematic/hazardous substance use in the absence of SUD.

Despite a high risk for the development of mental health disorders, refugees have legal access to just very restricted areas of general health care in Germany (36) and, regarding mental health care in receiving countries, face numerous structural and socio-cultural barriers that limit accessibility and utilization of services (37).

Digital health interventions (DHI) can address some of these barriers and can thus have an impact on the delivery of addiction prevention or harm reduction to marginalized populations. Structurally, DHI have the potential to reduce barriers such as a lack of resources, language barriers, and bureaucracy. As an example, Calvo et al. (38) demonstrated the accessibility of a smartphone app aimed at reducing harm associated with intravenous drug consumption among homeless drug users. Socio-culturally, DHI work with a high level of privacy and can reduce fear of stigma and discrimination (39), which would otherwise represent major barriers for individuals with substance use problems to seeking treatment (40). Furthermore, they may lower the impact of shame on utilization of services and bypass the necessity to ask for help personally; all of which were commonly seen as barriers to accessing traditional mental health care in Syrian refugees (37).

The majority of Syrian and Iraqi refugees in Germany have had access to a smartphone during migration (78 and 87%, respectively), do so upon arrival in Germany (89 and 92%, respectively), and use the internet daily (86 and 82%, respectively) (41). Therefore, we assume a high accessibility of smartphone-based DHI, e.g., apps, for the present study's target group. This, however, under two assumptions: first, that Afghan, Iranian, Iraqi, and Syrian refugees in Germany show a similar behavior toward apps and their usage in terms of needs and concerns as the German population. Second, that after cultural adaptations, content meets these needs also in terms of values, worldview, and cultural customs of the targeted population (42, 43). Otherwise, new barriers to accessing digital health care may arise (e.g., lack of technical literacy, difficulties accessing the internet, a doubt in the credibility, or low acceptance of services) (44). In terms of effectiveness, DHI have shown to be effective in reducing problematic use of alcohol compared to either no intervention or merely providing general health information and show similar effects as face-to-face interventions (45). The clinical significance for stand-alone and thus purely self-guided DHI is less clear compared to blended and therapist-guided digital approaches (46). As a strength—however—they may present a viable approach to the lack of language skills and cultural competencies of clinicians, but most importantly to lacking legal access to mental health care and unclear cost coverage for refugees in intermediate or final receiving countries, e.g., Germany. This leaves evidence-based stand-alone DHI a scalable and low-threshold service in reaching marginalized populations, such as refugees, who would face difficulties to make use of mental health services otherwise.

### Objectives

This study protocol describes the development and evaluation of a digital health intervention (DHI) for indicated prevention of SUD in young refugees in terms of its acceptance and usability in a single-armed feasibility trial. Additionally, the initial efficacy in reducing problematic use of alcohol and/or cannabis will be assessed by comparing pre- and post-intervention prevalence measures. As intervention, all participants will have access to the BePrepared App, which is an evidence-based stand-alone DHI optimally to be used in the participant's mother tongue and therefore available in Arabic, English, German, Farsi, and Pashto.

## Methods

We (1) developed and (2) plan to evaluate a digital health intervention (DHI) for the reduction of problematic substance use for young refugees from Afghanistan, Iran, Iraq, and Syria, aged 18 to 28 years. The targeted group shows signs of problematic use of alcohol and/or cannabis (see *Assessments* for details on the assessment of problematic substance use during the screening) and currently lives in Germany. This study protocol describes both steps (i.e., development and evaluation) following the recommendations of the SPIRIT 2013 statement [Standard Protocol Items: Recommendations for Interventional Trials; (47)].

### Development of the Digital Health Intervention

#### Theoretical and Clinical Framework

The digital health intervention's aim is the reduction of problematic substance use. The intervention is technically implemented in form of a smartphone application for stand-alone use. Overall, we aimed to develop a smartphone application that is easily understandable, rewarding (including positive reinforcement and feedback about progress), and beneficial [such as proposed by Crane et al. (48) for apps aiming at reducing excessive alcohol use]. The intervention development is further based on the IDEAS (Integrate, Design, Assess, and Share) framework for developing digital health behavior change interventions by Mummah et al. (49) as it may apply to the development of a digital behavior change intervention and its evaluation in a feasibility trial. The IDEAS framework consists of 10 phases: (1) empathize with target users, (2) specify behavior, (3) ground in behavioral theory, (4) ideate implementation strategies, (5) prototype potential products, (6) gather user feedback, (7) build minimum viable product, (8) pilot potential efficacy and usability, (9) evaluate efficacy in RCT, (10) share intervention and findings. In line with phase 1 and 6 of IDEAS, we took the following steps to ensure user-involvement with the hard-to-reach target group and to uncover needs, desires, and motivations of the target users:

1. Conduction of two focus groups [one with refugees of the targeted countries of origin (*n* = 7) and one with experts from the field of refugee and addiction services (*n* = 13)],
2. Consultation with professional cultural mediators (*n* = 5),
3. Consultation with the advisory board of refugees of the “PREPARE research consortium on the Prevention and Treatment of Substance Use Disorders in Refugees,”
4. Pilot-testing including interviews with young refugees (*n* = 9) gathering user feedback.

The conceptual design of the intervention (phase 3) is based on principles of cognitive behavioral therapy and implements selected behavior change techniques [following Michie et al. (50) taxonomy, see Table 1]. In an interdisciplinary team of psychologists, computer scientists, and app designers, the conceptual design, technical implementation, and app development took place in an iterative approach (phases 4, 5, and 7). Due to the study design, phases 2, 9, and 10 were skipped. For details on phase 8, please see *Evaluation of the digital intervention*.

**Table 1:** Content of the digital intervention.

**Module**	**Purpose**	**Exercises**	**Behavior change techniques incorporated [2]**
**Personalized feedback [1]** (see Figure 1)	• Informing about the user's substance use • Promote responsibility and self-efficacy • Referral to addiction treatment facilities in case of indications for alcohol or cannabis use dependence		• Provide information on social norms • Personalized normative feedback (comparison to people of same age and gender in the German population) • Motivational Interviewing techniques
**Diary** (main module 1, see Figures 2C, 3)	• Enhancing self-monitoring • Encouraging to reflect on substance use and situations in which one uses • Giving insight in the amount of pure alcohol (g) of different beverages	• Substance use diary	• Prompt self-monitoring of behavior • Foster identification of cues
**Goals** (main module 1)	• Introducing realistic goal setting	• Personalized goal-setting	• Prompt specific goal setting • Prompt review of behavioral goals • Provide feedback on behavior based on principles of Motivational Interviewing
**Changing** (main module 2, see Figure 4)	• Breaking down behavioral choices into the underlying motives • Reflecting on values	• Motivational “Scale” • “Values”	• Pros and cons of consumptions vs. non-consumption • Identification of incompatibility between beliefs and behavior • Prompt barrier identification • Feedback on motives based on principles of Motivational Interviewing • Prompt intention formation
**Thinking and Doing** (main module 3)	• Practicing to reject substance use • Activity planning • Activation of resources	• “No, thank you” • “Positive thoughts” • “Planning activities”	• Skills training with focus on thoughts and behavior • Rejection training • Prompt self-talk • Behavioral activation
**Relaxation** (main module 4, see Figure 5)	• Informing about emotions and their purpose • Learning how to regulate emotions • Calming down body and mind by breathing	• How to deal with sadness, anger, fear and nightmares • Breathing exercise	• Skills training with focus on emotions and behavior • Psychoeducation on emotions and its purpose • Monitoring of emotional effects • Feedback on outcomes of breathing exercise
**Knowledge** (optional module)	• Enhancing knowledge on alcohol and cannabis, its use, its consequences, the German culture, the German legal system	• “Good to know” • Quiz	• Provide information about behavior-health link • Provide information about health, social and emotional consequences • Provide instruction on safer use • Provide information on social support (practical) • Foster identification of self as a role model
**Treasure chest** (optional module, see Figure 6B)	• Encouragement to use the diary • Encouragement to reach goals • Providing an overview of successes, feedback and highlights	• “Successes” (Badges) • Feedback • “Moments” • Incentives	• Activation of resources • Provide contingent rewards • Provide general encouragement
**Help** (optional module, see Figure 6A)	• informing about the care system • Creating contact to help offers online or in person		• Provide information on social support (practical)
**Why?** (optional module, see Figure 6C)	• Transparency: Informing about the purpose of the exercises • Weaken doubts • Enhancing motivation		• Provide information about behavior-health link • Provide information on consequences of the exercises • Provide instruction on the exercises

*1: following Kamya's (51) FRAMES model; 2: taxonomy following (52)*.

#### Structure and Content of the Digital Intervention

**Figure 1:**
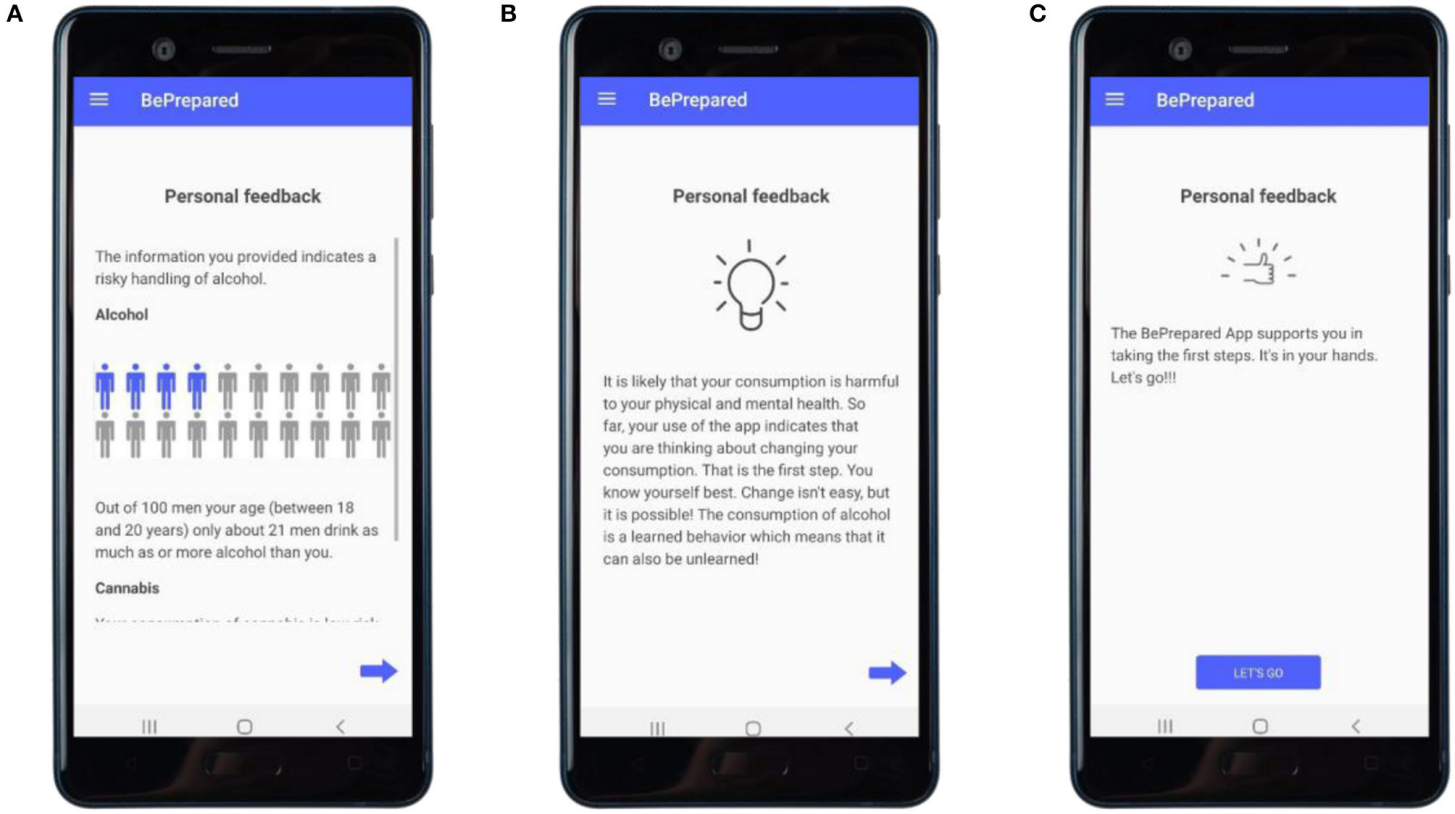
Personalized feedback. **(A)** Feedback shown to a male user, aged 18–20, with problematic use of alcohol using a subjective normative approach; screen 2 out of 4. **(B,C)** Feedback based on the FRAMES-Model (51); screen 3 and 4 out of 4.

**Figure 2:**
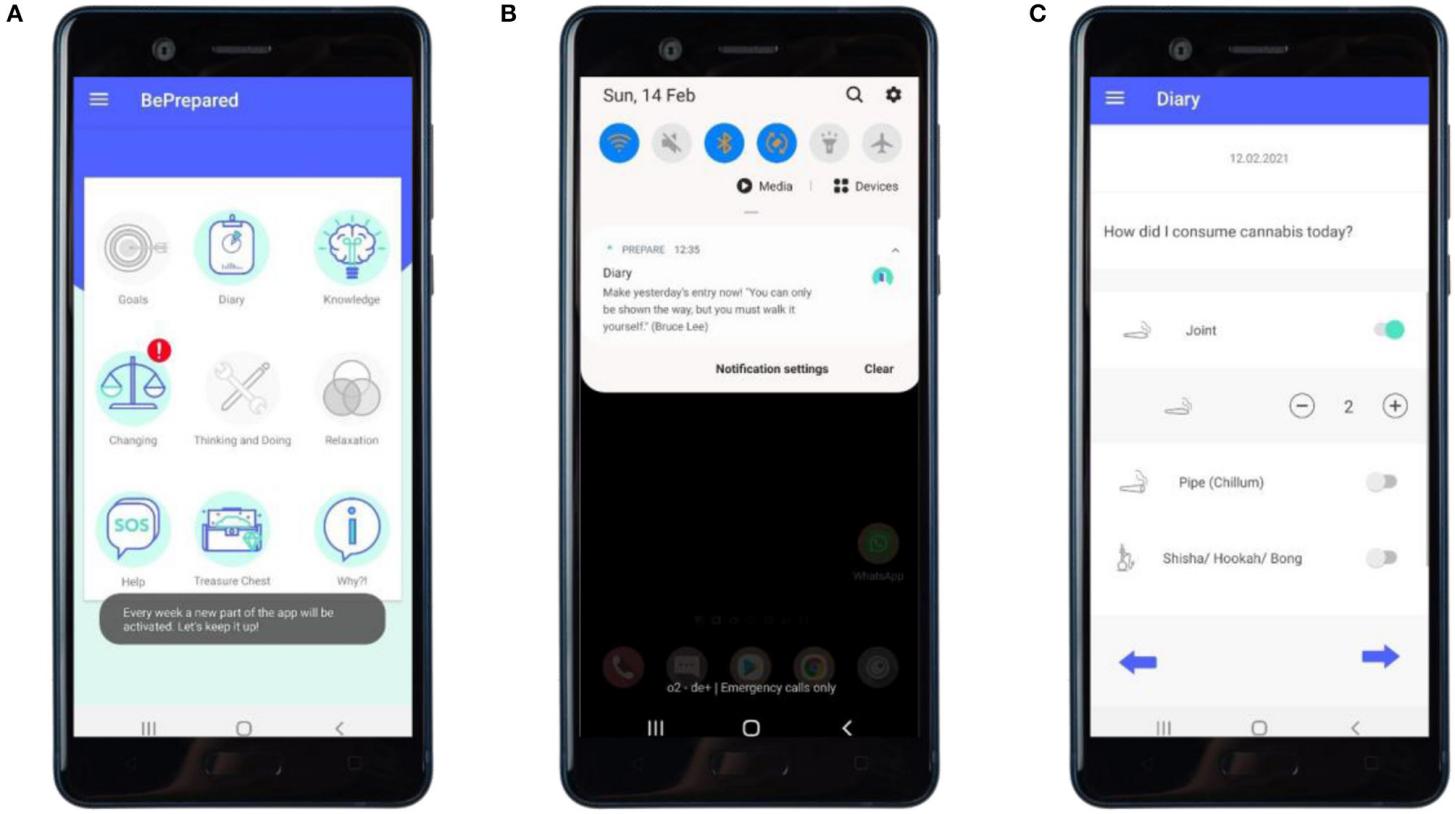
“Diary”: self-monitoring and prompts. **(A)** Main screen of the app with newly activated modules highlighted by alert and an explanatory toast regarding not yet activated modules. **(B)** Encouraging prompt on the user's home screen. **(C)** User making diary entry.

**Figure 3:**
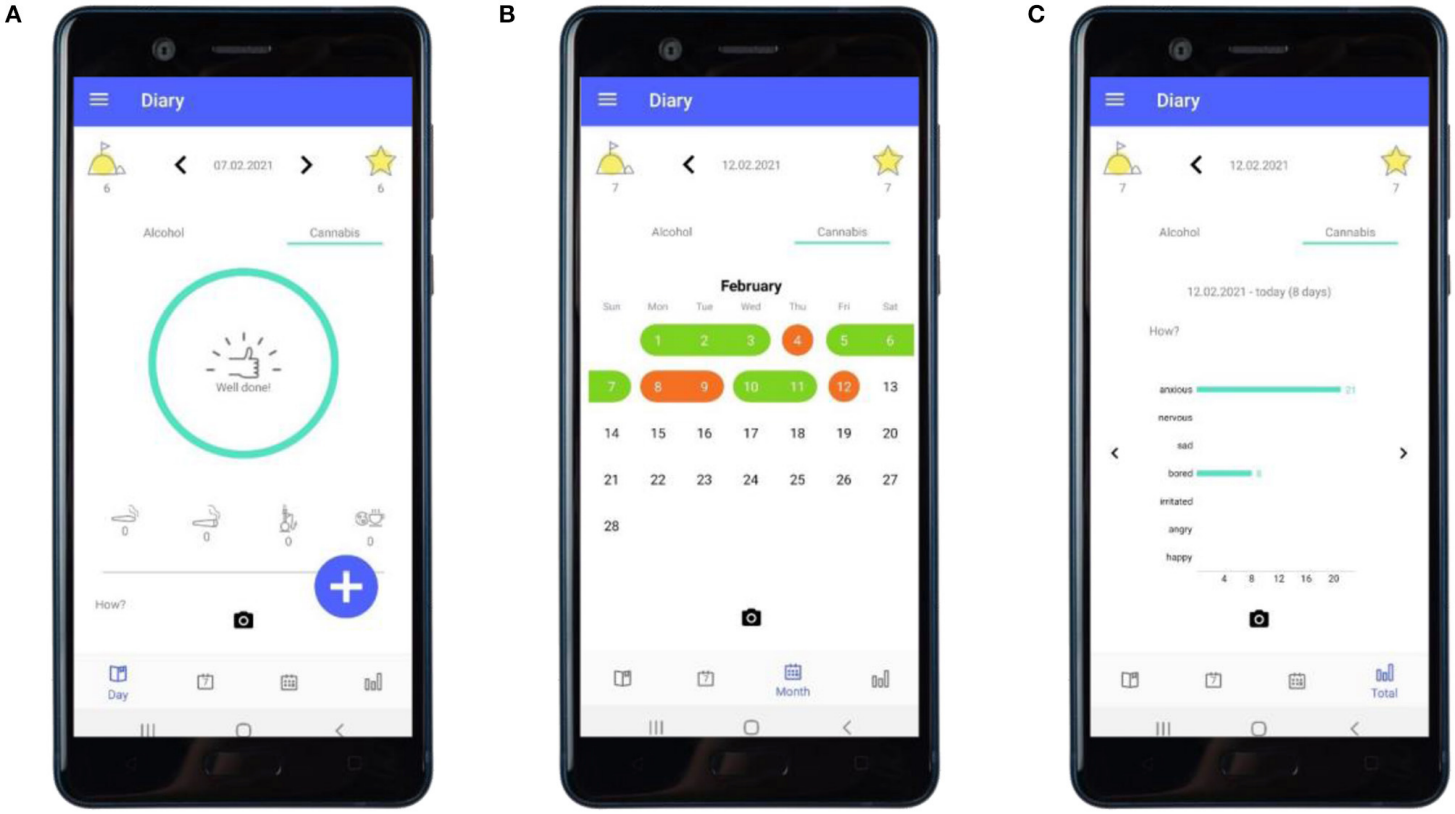
“Diary”: feedback about consumption. **(A)** Feedback following an entry of a day not having used cannabis. **(B)** The calendar providing an overview of a user's recorded use of cannabis. **(C)** The statistics section providing an overview of the user's recorded mood while having used cannabis.

**Figure 4:**
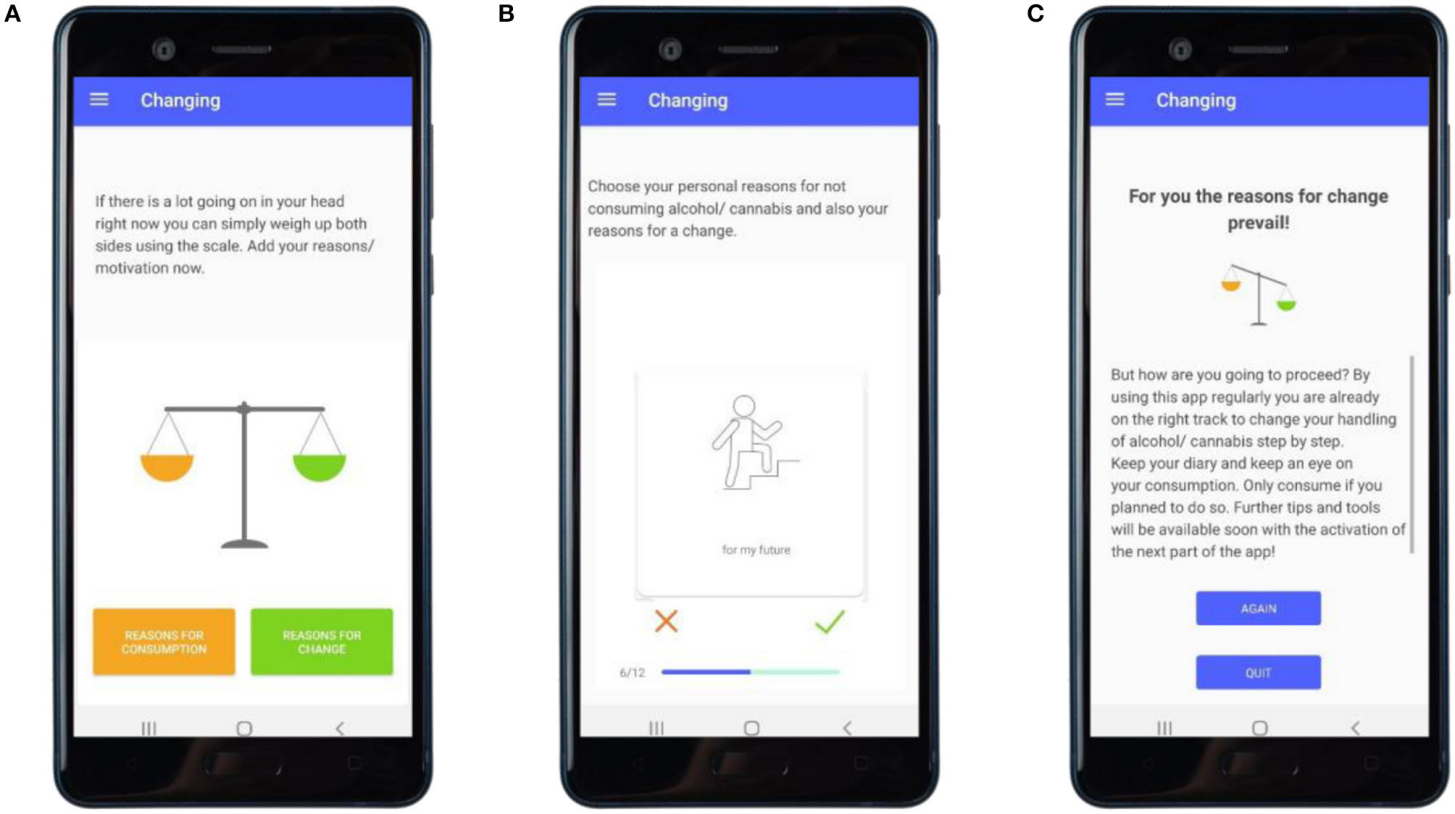
“Changing”: promoting motivation to change. **(A)** User has reflected on reasons for the use of substances and reasons for reducing the use of substances and starts to balance these in the motivational scale exercise. **(B)** User choses reasons for the use of substances and reasons for reducing the use of substances. **(C)** User receives feedback on personal reasons for substance use and reasons for changing one's substance use behavior.

**Figure 5:**
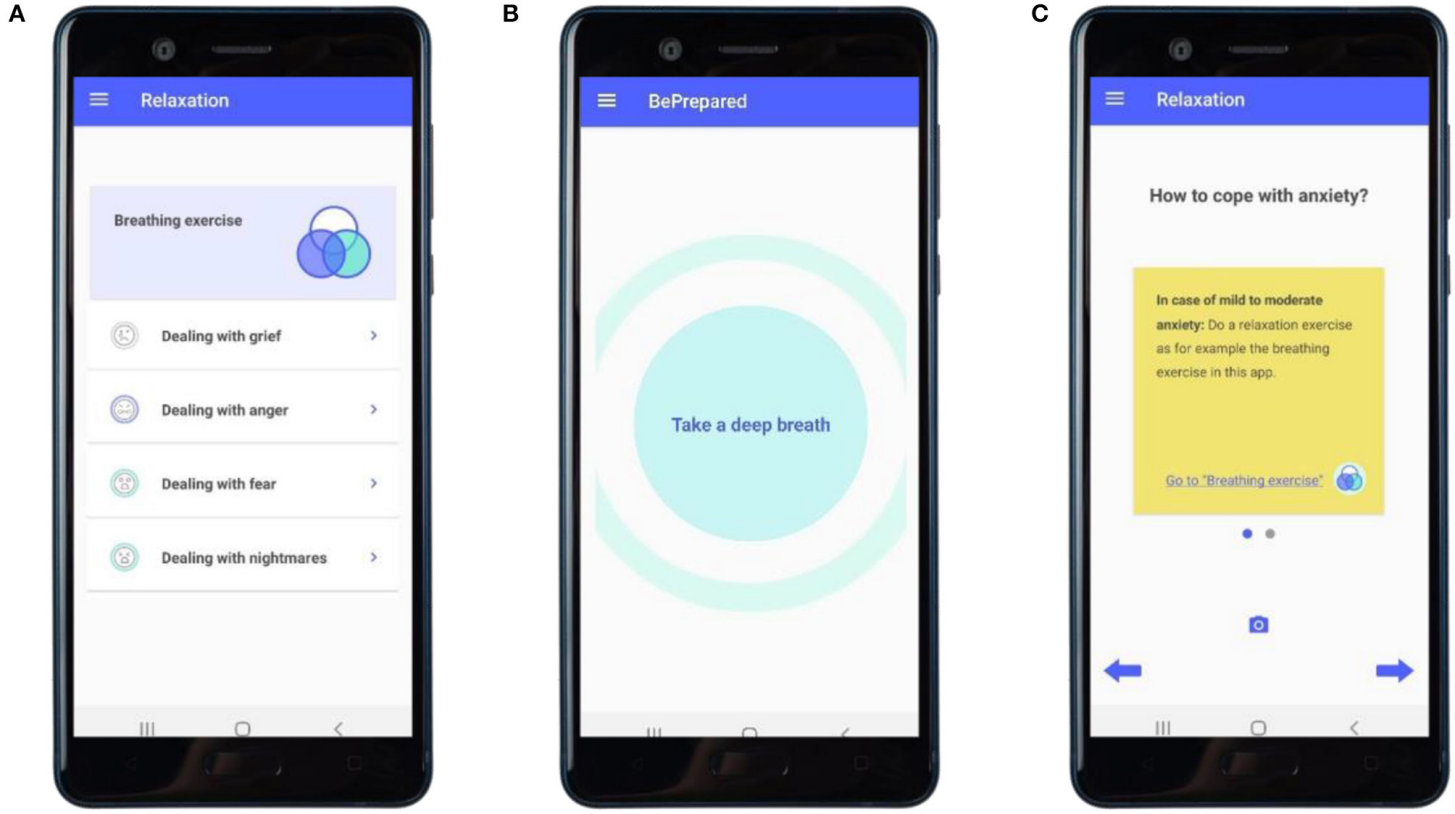
“Relaxation”: skills-training with focus on emotions and behavior. **(A)** Overview of the exercises on breathing, and dealing with grief, anger, anxiety, and nightmares. **(B)** Examplary screen of the breathing exercise. **(C)** Examplary advice on how to cope with anxiety.

After baseline assessment, the intervention starts with all study participants receiving a digital personalized feedback (see Figure 1) on their substance use following a subjective normative approach (53) and the FRAMES (Feedback, Responsibility, Advice, Menu Options, Empathy, and Self-Efficacy) model by Kamya (51). Four main modules are unlocked every week in four consecutive weeks (see Figure 2A for a visualization of newly activated modules). These main modules include: (1) promoting self-monitoring via a substance use diary (see Figure 2C) and feedback in accordance with previously made entries (see Figure 3); (2) promoting motivation to change (see Figure 4); (3) promoting skills focusing on behavior and thoughts, and (4) promoting skills focusing on emotions including a breathing exercise (see Figure 5). Three further modules encompass psychoeducation (i.a. on safe handling of alcohol and cannabis, advice on substance use reduction, and legal regulations), activation of personal resources, and information on the addiction help system in Germany. These modules can be used optionally. During the 4-week intervention phase, participants can use the activated app content according to their own needs. Pending follow-up measurements will block further use of the app (see *Assessments*). For an overview of the modules, see Table 1.

##### Referral Pathways

**Figure 6:**
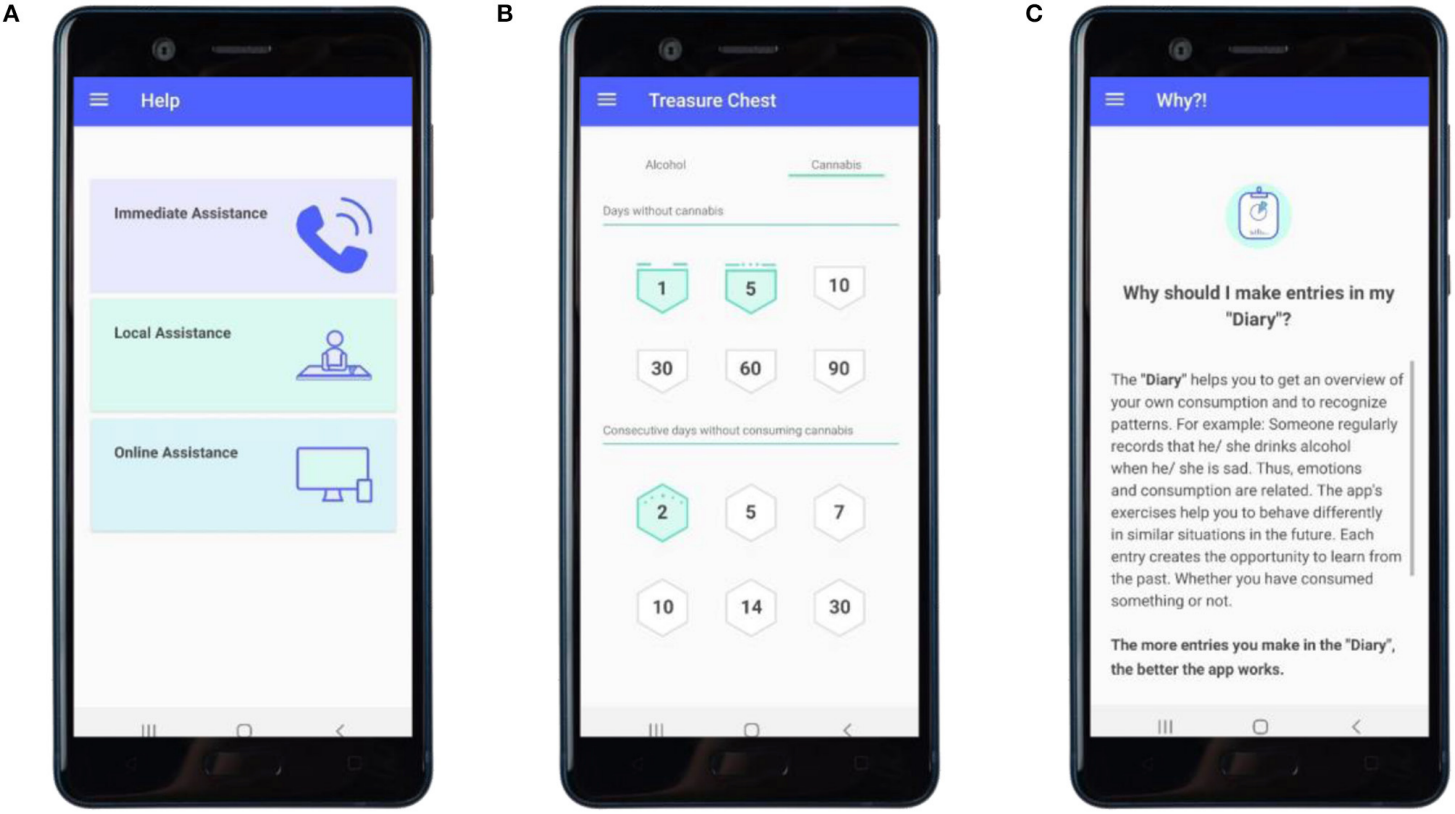
Further modules. **(A)** Overview of referral pathways offered in the app. **(B)** Overview of badges acquired. **(C)** Example of explanatory content on the intervention exercises.

Participants with high scores on the AUDIT or the CUDIT-R that indicate an alcohol or cannabis use dependency, respectively, will receive personalized feedback that suggests and encourages to seek help, provides an example for a counseling center and, in the case of high alcohol use, the warning to “not stop consumption without professional help as it could lead to physical harm or danger (e.g., cramping).” Furthermore, the intervention provides information on addiction counseling centers in general and highlights reasons to seek support based on the criteria of ICD-10 (54). In a separate module (see Table 1; Figure 6A) a number to call a hotline for immediate assistance in crisis situations, selected counseling centers and a link to a further (German-wide) search list for addiction counseling centers, as well as additional relevant online applications, are provided.

##### Cultural Adaptations

Critical to the development of the digital health intervention was the implementation of a number of culturally adapted elements. It follows Bernal and Sáez-Santiago's (42) guidelines on the practice of culturally competent psychology and is developed under consideration of both surface and deep structure dimensions (55). In line with these frameworks, the aspects of language, metaphors, the content, the concept, goals, methods, and the context as well as social, environmental, and psychological factors were considered when culturally adapting the intervention. The goal is to reduce (language) barriers and to maximize usability and acceptance of the intervention. The evaluation of above named focus group and consultation meetings with refugees and cultural mediators of the targeted countries of origin and experts from the field of refugee and addiction services lead to adapting single elements of the intervention:

The overall content of BePrepared has been formulated in easy language and translated into Arabic, English, German, Farsi, and Pashto by cultural mediators. These were instructed to translate content freely, e.g., sayings (module 3, see Table 1), metaphors, and concepts of distress (module 4, see Table 1), and in a spoken dialect rather than exact and possibly less comprehensible translations.

In a focus group with young refugees, we sketched a collection of motives for using and for not using substances and have integrated these in similar wording into the motivational scale exercise (module 2, see Table 1), a Motivational Interviewing technique (56).

In meetings with cultural mediators, we have e.g., identified values of the targeted culture (included in module 2, see Table 1), traditional sayings (implemented in the push notifications and module 3, see Table 1), adjusted wording, e.g., choosing the heading *positive thoughts* over the initial choice (*power-sentence)* or verbal ways to reject substance use in social situations (module 3, see Table 1), formulated suitable questions and answers implemented in an interactive quiz (based on the psychoeducative content, see Table 1), and adapted the information used for informed consent in terms of facilitated understanding.

In a focus group, experts from the field of refugee and addiction services highlighted the need for communicable data security, continuous reminders, a usage possible with limited internet connection and storage space, and a selection based on language skills rather than nationalities, which could all be implemented. The latter led to the separation of Group 1 (study participants) and Group 2 (app users), see under *Participants*.

The Arabic psychoeducative content was reviewed by an addiction counselor of Syrian origin to ensure that the thematic priorities had been remained after translation.

### Evaluation of the Digital Intervention

#### Study Design

We conduct a single-armed feasibility trial among 150 young refugees with problematic use of alcohol and/or cannabis in order to evaluate the intervention's usability and acceptability. Following the screening, participants will receive a digital health intervention (DHI) for the reduction of problematic substance use, introduced by a personalized feedback and carried out over a 4-week time frame. Follow-ups take place after 4 weeks (t_1_), 3 months (t_2_), and 6 months (t_3_).

#### Study Outcomes

We adopt a mixed methods approach to establish feasibility and acceptability of the DHI for the reduction of problematic substance use for young refugees, with data collected as outlined below.

#### Primary Outcomes

##### Feasibility

**Figure 7:**
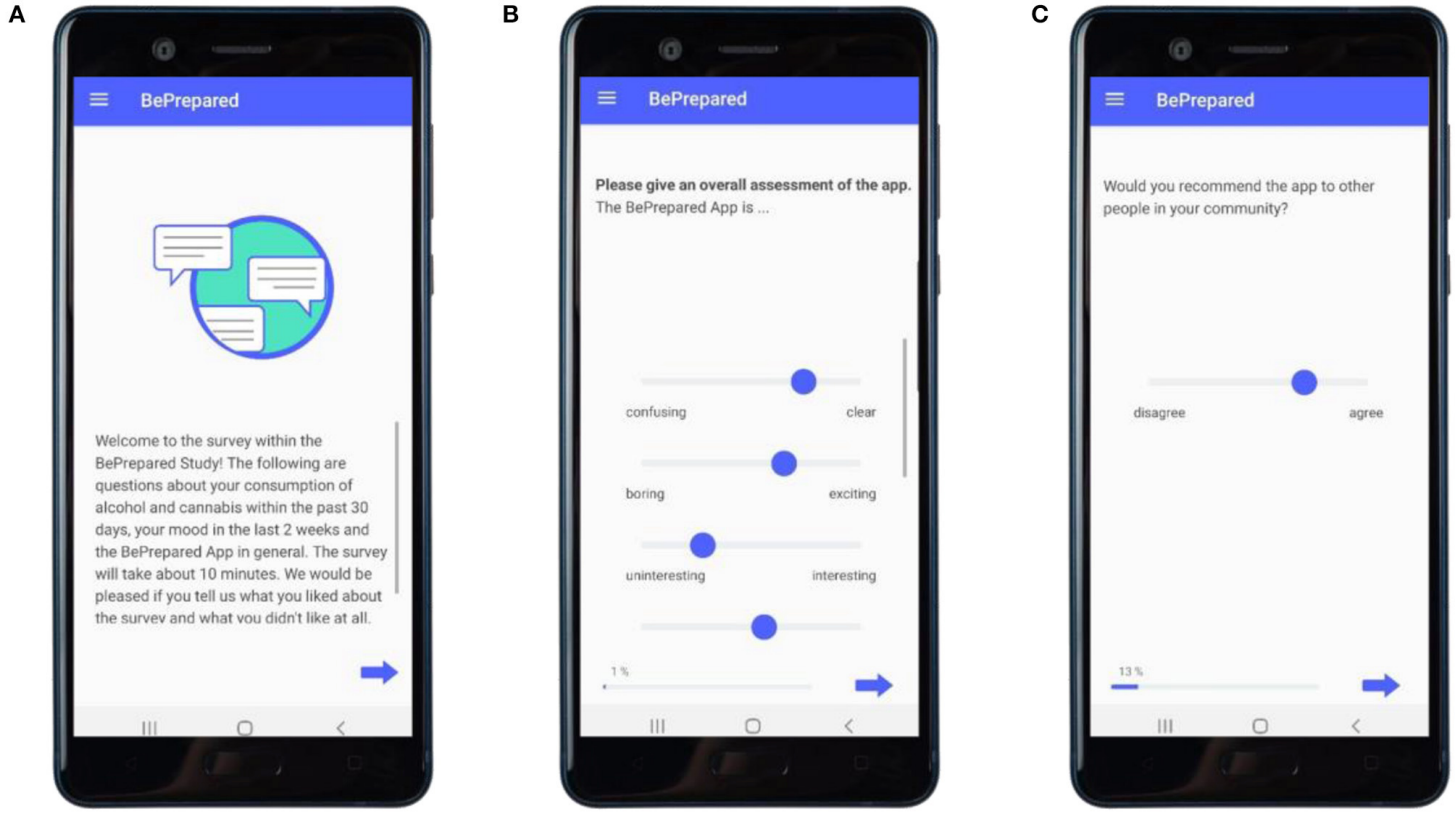
Implementation of the follow-measurements in the app. **(A)** Entry screen. **(B)** Examplary item of the overall assessment on the app. **(C)** Example item on cultural sensitivity.

The feasibility of the DHI for substance use reduction will be assessed using a self-constructed Programme Participation Questionnaire (PPQ) which you can find in full length in Appendix 1. See Figure 7 for an excerpt of its visualization in the application. Furthermore, we will quantitatively assess the use of the app through the app's meta-data log-files, collecting data on:

1. the rate of completion of the intervention (i.e., number of participants who access and complete the following aspects of the intervention: screening, feedback, minimum 10 min spent in three of the four main modules),
2. the frequency and duration of the intervention use,
3. the preferences in intervention use (i.e., modules of the smartphone intervention most frequently assessed and on which most time was spent), and
4. the number of times the smartphone app was assessed).

We hypothesize that the intervention is feasible and accepted by the target group.

##### Acceptability

Acceptability, perceived usefulness, and user-friendliness of the DHI and study procedures will be assessed using above named Program Participation Questionnaire. Quantitative data will be collected to assess the quality of each module and the overall cultural appropriateness of the DHI (see *Assessments*).

#### Secondary Outcome

As a secondary outcome, we will assess the initial efficacy of the DHI by the change from baseline in severity of substance use, depressive symptoms, and symptoms of anxiety. Comparing post-intervention to baseline levels, we will quantify

1. the initial efficacy of intervention using a 30-day prevalence measure in line with the Epidemiological Survey of Substance Abuse (2018),
2. the change in depressive symptoms with the Patient Health Questionnaire [PHQ-9; (57)], and
3. the change in symptoms of anxiety with the Generalized Anxiety Disorders Scale-7 (GAD-7; 54).

For an overview of all outcomes and associated measures, see Table 2.

**Table 2:** Measures and assessment at points used in the study.

**Construct (Assessment)**	**Baseline (t_**0**_)**	**Follow-up (t_**1**_/t_**2**_/t_**3**_)**	**Sources of translations**
Sociodemographics^a^^,^ ^b^			See note 2
Age	x		
Gender	x		
Home country	x		
Living situation	x		
Residence status	x		
Occupational status (past and present)	x		
Level of education	x		
Family status	x		
Information on children	x		
Religion	x		
Socio-economic status	x		
Additional inclusion criteria			Arabic, English, Farsi, Pashto: own translations
Refugee background	x		
No current psychiatric or psychological treatment for use of alcohol and/or cannabis	x		
Escape-related information			See Note 2
Dates of escapes' beginning^a^^,^ ^b^	x		
Duration of escape^a^^,^ ^b^	x		
Duration of stay in Germany^a^^,^ ^b^	x		
Accompaniment during escape^a^^,^ ^b^	x		
Burdens of missing information on family and friends^b^	x		
Substance use			
Alcohol (AUDIT)	x		Arabic: Almarri et al. (58); Farsi: Noorbakhsh et al. (59); German: Dybek et al. (60); Pashto: own translations
Cannabis (CUDIT-R)	x		Own translations
30-day prevalence	x	x	Own translations
Confidence in change	x	x	Own translations
Medical Questionnaire (pregnancy, serious disease, medication, SUD)	x		Own translations
Depression (PHQ-9)	x	x	German: Gräfe et al. (61); Arabic, Farsi, Pashto: own translation
Anxiety (GAD-7)	x	x	German: Löwe et al. (62); Arabic, Farsi, Pashto: own translation
Smartphone Use	x		own translations
Program Participation Questionnaire^b^		x	own translations

*1*:

a *Standardized measures with the PREPARE and PrevDrop research consortia*;

b *Standardized measures with the the KS-Diagnostik project*;

*^c^see attachments for full questionnaire*.

*AUDIT, Alcohol Use Disorders Identification Test; CUDIT-R, Cannabis Use Disorders Identification Test-Revised; PHQ-9, Patient Health Questionnaire; GAD-7, Generalized Anxiety Disorders Scale-7*.

*2: German, English, Arabic, and Farsi translations were shared by the research project PrevDrop on the Prediction and prevention of dropout in research, diagnostics, and treatment with refugees (contact: Thomas Ehring, Sekretariat.Ehring@psy.lmu.de, https://www.gesundheitsforschung-bmbf.de/de/prevdrop-vorhersage-und-pravention-von-dropout-in-forschung-diagnostik-und-behandlung-mit-9672.php). For Pashto, we use own translations*.

#### Participants

Study participants will be Afghan, Iranian, Iraqi, and Syrian refugees living in Germany. We will recruit participants with a multi-strategic approach in Germany (see *Recruitment*).

During the onboarding of the app, written informed consent will be obtained. A screening in form of an online questionnaire will determine up to *n* = 150 refugees for eligibility for study participation.

Inclusion criteria are:

1. age between 18 and 28 years;
2. country of origin being Afghanistan, Iran, Iraq, or Syria;
3. self-declared refugee background;
4. hazardous/problematic use of alcohol and/or cannabis determined as described below;
5. informed consent to participate in the study;
6. agreement with the application's terms of use.

Participants identified as eligible after study participation screening will be assigned to Group 1 (study participants). Participants that violate inclusion criteria (1) to (4), but are older than 18 years of age, to still be able to download and use the app and to be assigned to Group 2 (app users). This is in order to foster the dissemination of the app and to prevent systematic discrimination and potentially negative attitudes toward the app usage due to perceived unfairness. Minors are neither eligible for study participation nor app use.

Exclusion criteria for study participation are an age of under 18 years, a country of origin other than Afghanistan, Iran, Iraq, or Syria, no self-declared refugee background, no hazardous/problematic alcohol- and/or cannabis-use or negated consent to the study participation or the app's terms of use, and a current participation in an in- or out-patient treatment for SUD. For promoting dissemination, an exception is the participation in a group intervention program on substance use disorder and trauma (contact Ingo Schäfer at i.schaefer@uke.de for more information) which will be assessed during the screening and which takes place within the “PREPARE research consortium on the Prevention and Treatment of Substance Use Disorders in Refugees”.

#### Recruitment

We will apply a multi-strategic approach to recruit Syrian, Iranian, Iraqi, and Afghan refugees living in Germany. First, our strategy includes training keypersons (e.g., people working in refugee accommodation centers, information centers, psychosocial centers, addiction counseling centers, …). Keypersons are trained in a workshop on how to address members of the targeted group in person, according to principles of Motivational Interviewing (56). The workshop is complemented by a booklet, which can be found for download in German language at: https://www.katho-nrw.de/fileadmin/primaryMnt/KatHO/DISuP/BePrepared/Praxisbuch_201104.pdf. Second, we will adopt a snowball sampling approach, communicated to the targeted group by the trained keypersons, which suggest refugees to spread information on the study within their personal social network and community. Third, we will distribute multi-lingual advertising material (i.e., posters and flyers) in facilities frequented by refugees. Fourth, multiple online platforms (e.g., the webpages www.beprepared-app.de, www.sucht-und-flucht.de, Google Play Store …) will advertise the study and app. The download of the app via the Google Play Store (see https://play.google.com/store/apps/details?id=dai.com.prepare) will be free of charge, however, restricted to users currently residing in Germany. A list of study sites is available on the following webpage: https://www.katho-nrw.de/katho-nrw/forschung-entwicklung/institute/disup/forschungsprojekte/beprepared/.

#### Assessments

**Figure 8:**
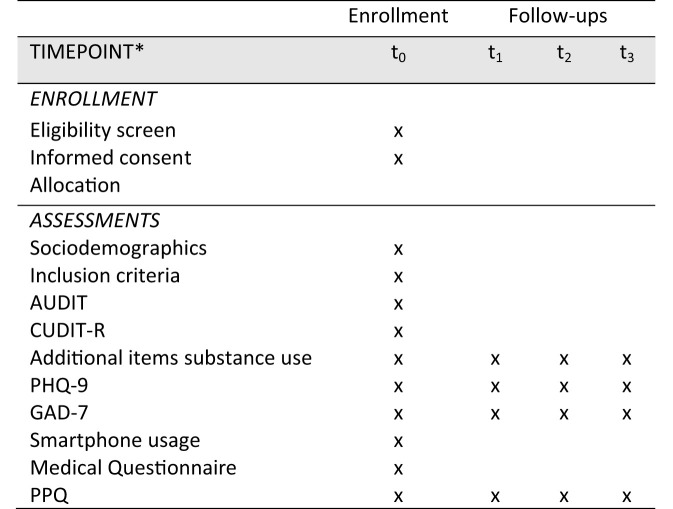
Schedule of enrollment, interventions and assessment. Completed SPIRIT 2013 figure of recommended content of enrollment, interventions, and assessments. t_0_, baseline; t_1_, 4 weeks after baseline (post intervention); t_2_, 16 weeks after baseline; t_3_, 24 weeks after baseline.

After obtaining informed consent and an eligibility screen on the app usage (minimum of 18 years of age), the baseline-measurement (t_0_) is scheduled. The baseline questionnaires will consist of items relating to sociodemographics, escape related information, a medical questionnaire on pregnancy, serious disease, medication and SUD, depression, anxiety, and smartphone use (see Table 2). Follow-up measurements are scheduled directly after the intervention (t_1_), as well as at 3-months (t_2_), and at 6-months (t_3_) follow-ups. Assessment and intervention take place on the participants' mobile devices and pending assessments will block further use of the app. A schedule of enrollment, intervention and assessment is shown in Figure 8.

To assess problematic use of alcohol and/or cannabis, all participants receive a screening using the Alcohol Use Disorder Test [AUDIT; (63)] and a from 6- to 12-months adapted version of the Cannabis Use Disorder Test-Revised [CUDIT-R; (64)] at baseline t_0_. The AUDIT is a 10-item screening tool that assesses individual alcohol consumption, with scores for each question ranging from 0 to 4 (e.g., *never* scoring 0, e.g., *less than monthly* scoring 1, e.g., *monthly* scoring 2, e.g., *weekly* scoring 3, and e.g., *daily or almost daily* scoring 4) and overall scores ranging from 0 to 40. Scores of 0 to 5 and 0 to 4 indicate a safe use for males and females, respectively, scores from 5 to 19 and 4 to 19 indicate problematic use for males and females, respectively, and scores of 20 or higher is likely to indicate alcohol dependence. The CUDIT-R consists of eight items, screens individual cannabis consumption, with scores ranging from 0 to 4 (e.g., *never* scoring 0, e.g., *less than monthly* scoring 1, e.g., *monthly* scoring 2, e.g., *weekly* scoring 3, and *daily or alm*ost daily scoring 4). For all genders, a score of ≥ 8 and <12 represents problematic use of cannabis. The Generalized Anxiety Disorders Scale-7 [GAD-7; (65)] assesses symptoms of anxiety and consists of seven items, each scored from 0, *not at a*ll, to 3, *nearly every day*. Despressive symptoms are assessed with the Patient Health Questionnaire [PHQ-9; (57)], a screening tool that consists of nine items, as well as the GAD-7 items each scoring from 0, *not at all*, to 3, *nearly every day*. For the sources of translations see Table 2.

##### Study Adherence

In order to lower the risk for dropout, participants will be informed on the study procedure including the time needed to complete the digital health intervention and the follow-up measurements, and on incentives. Group 1 will receive a €15 gift card as a reward for their participation after completing measurement t_1_, t_2_, and t_3_. Group 2 will participate in a tombola with a minimum one in ten chance of winning a €15 gift after completing the measurements at t_1_, t_2_, and t_3_.

There are regular reminders in form of push notifications with the goal to draw attention to unused intervention components, missed diary entries, and the follow-up measurements (see Figure 2B). Study adherence will be assessed through the app's meta-data log-files which give information on the frequency and duration of intervention use.

#### Sample Size

In this Stage I clinical feasibility trial the emphasis lies on examining feasibility and acceptability of the intervention as well as on a first preliminary estimate of the intervention's efficacy. For this purpose a sample size of at least *n* = 150 is targeted. To be assessed for eligibility: *n* = 450; to be allocated to trial: *n* = 150; to be analyzed at t1: *n* = 150. While there is little to no literature on dropout rates in refugee populations (66), a literature review by Swift and Greenberg (67) shows highly heterogenous dropout rates in studies on psychological treatment, ranging from 0 to 74.23%, with a weighted drop out rate of 19.7%. Regarding digital interventions, a review of internet-based treatment for psychological disorders shows an average dropout of 31% (ranging from 2 to 83%) (68) and a more recent systematic review and meta analysis on studies of apps for depressive symptoms shows dramatic dropout rates of almost 50% when accounting for bias (69). Hence, the present intervention's dropout rate istself will be a strong indicator for the study's feasibility.

#### Statistical Analysis

Qualitative and quantitative descriptive data analyses will be conducted according to the research questions. Confidence intervals will be determined for both the frequency of use of the main modules and the completion rate of the intervention until t_1_. We will investigate change in the 30-days prevalence of alcohol and/or cannabis and on the GAD-7 and the PHQ-9 scores using linear mixed effect models.

#### Ethical Considerations

Before the trial, ethical approval was obtained from the ethics board of the Catholic University of Applied Sciences North Rhine-Westphalia. All data processing will be conducted according to the current Data Protection Act, and the data protection officer of the above-named university monitors issues regarding data protection. All study participants and app users will receive detailed information on study goals, analyses, and data reporting. They will give their informed consent and accept the terms of use of the application before participation in the study. All data are saved on a protected server.

Serious Adverse Events (SAE; pregnancy, diagnosed addiction, sickness, heart or liver disease) are considered to be theoretical and are not expected. However, these events are registered using a medical questionnaire, which provides feedback on health-related contraindications regarding any further substance use. This information is systematically documented throughout the assessments.

#### Data Monitoring

The PREPARE research consortium (“Prevention and Treatment of Substance Use Disorders in Refugees (PREPARE)” organizes a central data safety and monitoring board. A list of members can be requested at the consortium coordinator Prof. Dr. Ingo Schäfer (i.schaefer@uke.de).

## Discussion

The present study protocol describes the development and the design of the evaluation study on the “BePrepared App” as a digital health intervention (DHI) for a substance use reduction in young refugees with problematic use of alcohol and/or cannabis. The primary goal of the feasibility trial is to assess the DHI's usability and acceptability among the targeted group. Secondary, we aim to give an indication on its initial efficacy in leading to a substance use reduction. According to our preliminary research and a rapid review by Liem et al. (70), which identified 16 digital mental health technologies for immigrants and refugees, however, none of which targeting substance use prevention or treatment, we believe the “BePrepared App” to be one of the first digital health intervention to deal with the indicated prevention of substance use disorder among refugee populations worldwide and to be scientifically evaluated (71). If successful, this study may contemplate to overcoming care barriers by providing refugee populations with urgently needed access to preventive health care through delivering accessible indicated prevention. As a stand-alone intervention not presenting personalization or face-to-face contact, it may become a significant additional tool within a larger set of interventions (72).

### Implications

This evaluation study allows to draw conclusion about the usability and acceptability of the DHI among young refugees with problematic use of alcohol and/or cannabis. This forms the basis for further research on the efficacy of the DHI. The description of the intervention development and study design allows to replicate the study as well as to retrace where the weaknesses and strengths of the DHI lie.

Under the assumption that the DHI is in fact feasible in terms of being used and accepted by the targeted group, the DHI itself has several strengths. It is independent of location and time, free of costs, and easily accessible. It reduces language barriers, which in turn reduces treatment barriers. It addresses the topic of substance use and substance use prevention with cultural sensitivity and respects the target users' social contextual factors.

### Limitations

Concerning the development of the DHI, we did not have the resources to apply a fully iterative co-design [such as e.g., Martin et al. (73)] or community-driven adaptations of an existing intervention [such as e.g., (74)]. Translating the application from German into Arabic, English, Pashto, and Persian and involving cultural mediators and refugees from a number of countries (Afghanistan, Iran, Iraq, and Syria) in the intervention development did not allow to limit cultural adaptations to one cultural group and may weaken the possible benefits of cultural adaptations compared to if done so. Furthermore, limited resources did only allow to review Arabic translations by an addiction counselor of Syrian nationality (Arabic speaking) to ensure that the thematic priorities had been remained after translation.

In this pilot stage, randomized allocation to different groups is not feasible and exceeds the resources of this study. Thus, we will not apply a control group in this feasibility study, and consequently, no treatment comparisons are tested. We will estimate the effect sizes of the initial efficacy as a secondary endpoint, which, however, does not give high-quality evidence. Furthermore, the application of a non-probability sampling technique will not allow generalization. Future research may compare the treatment effects of this DHI in comparison to absence of, minimal, or face-to-face interventions using a control group and randomization. Bias regarding systematic dropout is not to be fully excluded in this single-armed trial. Furthermore, due to having fled political or religious persecution, participants of specific ethnic backgrounds may be reluctant to share personal data and be hesitant to trust the data safety policies operated in this study. Along with varying rates of illiteracy in the target population, this reluctance may create a selection effect in the sample. Since no personal contact is established, no rejections of the DHI before study participation can be documented.

## Ethics Statement

The studies involving human participants were reviewed and approved by the ethics committee of the Catholic University of Applied Sciences North Rhine-Westphalia in Cologne, Germany, approved all procedures. All procedures in the study adhere to Good Clinical Practice (GCP) standards, including the written informed consent from all participants. Before participants are included in the study, they are informed about the aims and the design of the study and the possibility of ending their participation at any time without disadvantage. The patients/participants provided their written informed consent to participate in this study.

## Author Contributions

LF, VK, and MK were responsible for the intervention development. LF wrote the first draft of the manuscript. VK, NW, and MK corrected and complemented the manuscript. NW and MK designed the study and obtained funding for the project. All authors read and approved the final manuscript.

## Conflict of Interest

The authors declare that the research was conducted in the absence of any commercial or financial relationships that could be construed as a potential conflict of interest.

## Abbreviations

AUDIT: Alcohol Use Disorder Identification Test
CUDIT-R: Cannabis Use Disorder Identification Test-Revised
GAD-7: Generalized Anxiety Disorder Scale-7
MC: Multiple Choice
PHQ-9: Patient Health Questionnaire-9 (PHQ-9) depressive symptoms
SAE: severe adverse event
PPQ: Programme Participation Questionnaire
SC: Single Choice
SES: Socioeconomic status
SUD: substance use disorder
VAS: Visual Analog Scale.
