# Supplementary material for: Development and Evaluation of a Digital Health Intervention for Substance Use Reduction in Young Refugees With Problematic Use of Alcohol and/or Cannabis—Study Protocol for a Single-Armed Feasibility Trial
Source: Front Public Health. 2021 Mar 31;9:557431. doi: 10.3389/fpubh.2021.557431 (PMC8044446; doi:10.3389/fpubh.2021.557431)
Supplement: Supplementary file 1 [file Data_Sheet_1.docx]

| **Appendix 1** Self-constructed Program Participation Questionnaire (PPQ) | | | | | | |
| --- | --- | --- | --- | --- | --- | --- |
|  | **Item** | |  | **Answer options** |  | **Answer type** |
| 1 | Please give an overall assessment of the app. The BePrepared App is … | |  | confusing/clear; boring/exciting; uninteresting/interesting; irrelevant/relevant; unappealing/appealing; demotivating/motivating; short/long |  | VAS |
| 2 | Please give an overall assessment of the app. What did you like best about the app? [multiple answers possible!] | |  | list of sections, exercises and functions of the app^a^ |  | MC |
| 3 | Please give an overall assessment of the app. What did you like least about the app? [multiple answers possible!] | |  | list of sections, exercises and functions of the app^a^ |  | MC |
| 4 | The design of the app is appealing. | |  | strongly disagree/strongly agree |  | VAS |
| 5 | The wording of the app is engaging. | |  | strongly disagree/strongly agree |  | VAS |
| 6 | When I use the app in public, I feel … | |  | uncomfortable/comfortable |  | VAS |
| 7 | By using the app, I have … | |  | learned nothing/learned a lot |  | VAS |
| 8 | Where you able to use the acquired knowledge in your everyday life? | |  | not at all/very much |  | VAS |
| 8 | All in all, how do you like the app? | |  | not at all/very much |  | VAS |
| 10 | I would use the app again. | |  | strongly disagree/strongly agree |  | VAS |
| 11 | Would you recommend the app to other people in your community? | |  | not at all/very much |  | VAS |
| 12 | “Diary”^b^ |  |  |  |  |  |
|  |  | “Diary”. I had a look at the section. |  | strongly disagree/strongly agree |  |  |
|  |  | The section “Diary” is … |  | confusing/clear; boring/exciting; uninteresting/interesting; irrelevant/relevant; unappealing/appealing; demotivating/motivating; short/long |  |  |
|  |  | “Diary”. All in all: how do you like this section? |  | not at all/very much |  |  |
| 13 | Through the app I became aware of further offers of assistance on the subject of alcohol and / or cannabis- | |  | Yes/No |  | SC |
|  |  | If yes:  Through the app I used offers of assistance on the subject of alcohol and / or cannabis. |  | Yes/No |  | SC |
|  |  | If yes:  Which offers of assistance did you use (besides the BePrepared App)? |  | Addiction counseling center/general counseling center/online assistance on the subject of alcohol or cannabis (other apps or programs)/general online assistance (apps or programs on living in Germany)/”Immediate Assistance” (telephone counseling on alcohol and/or drugs) |  |  |
|  |  | |  |  |  |  |
| Abbreviations: VAS = Visual Analogue Scale; MC = Multiple Choice; SC = Single Choice  ^a^full list: Make entries in the “Diary”/Have a look at the overviews in the “Diary”/the knowledge quiz/the info texts “interesting facts”/the exercise “Scale”/the exercise “Values”/the exercise “No, thank you!”/the exercise “Positive thoughts”/the exercise “planning activities”/the breathing exercise/the section “Grief”/the section “Anger”/the section “Fear”/the section “Nightmares”/the section “Immediate Assistance”/the section “Local Assistance”/the section “Online Assistance”/the cumulative “Achievements”/the collected “Moments” (photo function)/the “Feedbacks”/the info texts “Why?!”/the reminders of the app/the highlighting of what is “important” to me (red exclamation marks)  ^b^ “Diary” is an example and exchanged by each section of the app. | | | | | | |
